# Microbially derived surfactants: an ecofriendly, innovative, and effective approach for managing environmental contaminants

**DOI:** 10.3389/fbioe.2024.1398210

**Published:** 2024-08-26

**Authors:** Navdeep Singh, Xiao-Hu Hu, Vikash Kumar, Manoj Kumar Solanki, Amit Kaushik, Vipin Kumar Singh, Sandeep Kumar Singh, Priya Yadav, Rahul Prasad Singh, Nikunj Bhardwaj, Zhen Wang, Ajay Kumar

**Affiliations:** ^1^ Department of Chemistry, N.A.S.College, Meerut, India; ^2^ Guangxi Key Laboratory of Agricultural Resources Chemistry and Biotechnology, Agricultural College, Yulin Normal University, Yulin, China; ^3^ Faculty of Agricultural Sciences, GLA University, Mathura, India; ^4^ Department of Life Sciences and Biological Sciences, IES University, Bhopal, India; ^5^ College of Biotechnology, Chaudhary Charan Singh Haryana Agricultural University (CCSHAU), Hisar, India; ^6^ Department of Biotechnology, Graphic Era (Deemed to be University), Dehradun, India; ^7^ Department of Botany, K.S. Saket P.G. College, Ayodhya, India; ^8^ Division of Microbiology, Indian Agricultural Research Institute, New Delhi, India; ^9^ Centre of Advanced Study in Botany, Banaras Hindu University, Varanasi, India; ^10^ Department of Zoology, Maharaj Singh College, Maa Shakumbhari University, Saharanpur, India; ^11^ Amity Institute of Biotechnology, Amity University, Noida, India

**Keywords:** biosurfactants, hydrophobic chemicals, interfacial surface tension, emulsifiers, environmental pollutants, circular economy

## Abstract

The natural environment is often contaminated with hydrophobic pollutants such as long-chain hydrocarbons, petrochemicals, oil spills, pesticides, and heavy metals. Hydrophobic pollutants with a toxic nature, slow degradation rates, and low solubility pose serious threats to the environment and human health. Decontamination based on conventional chemical surfactants has been found to be toxic, thereby limiting its application in pharmaceutical and cosmetic industries. In contrast, biosurfactants synthesized by various microbial species have been considered superior to chemical counterparts due to their non-toxic and economical nature. Some biosurfactants can withstand a wide range of fluctuations in temperature and pH. Recently, biosurfactants have emerged as innovative biomolecules not only for solubilization but also for the biodegradation of environmental pollutants such as heavy metals, pesticides, petroleum hydrocarbons, and oil spills. Biosurfactants have been well documented to function as emulsifiers, dispersion stabilizers, and wetting agents. The amphiphilic nature of biosurfactants has the potential to enhance the solubility of hydrophobic pollutants such as petroleum hydrocarbons and oil spills by reducing interfacial surface tension after distribution in two immiscible surfaces. However, the remediation of contaminants using biosurfactants is affected considerably by temperature, pH, media composition, stirring rate, and microorganisms selected for biosurfactant production. The present review has briefly discussed the current advancements in microbially synthesized biosurfactants, factors affecting production, and their application in the remediation of environmental contaminants of a hydrophobic nature. In addition, the latest aspect of the circular bioeconomy is discussed in terms of generating biosurfactants from waste and the global economic aspects of biosurfactant production.

## 1 Introduction

Rapid industrialization and incessantly increasing anthropogenic activities have raised the concern of multifarious contaminant release as well as accumulation in the natural ecosystems, thereby adversely affecting the ecosystem functioning and human livelihood (Santos B. L. P. et al., 2023; Kabir et al., 2023; Silva et al., 2024). Although conventional methods such as landfilling, incineration, and oxidation–reduction have been widely practiced to mitigate such challenges, especially in developing countries (Hu et al., 2021), the limited land resources, high costs, and the deposition of toxic chemical residues in the environment have raised questions about the traditional mitigation approaches (Bhatt et al., 2022). At the same time, organic contaminants of a hydrophobic nature, such as oil spills, petrochemicals, and long-chain hydrocarbons, have posed significant adverse impacts due to their toxic nature, insolubility, low degradation rate, and high persistence (Kalia et al., 2022; Shaji et al., 2024).

Recently, microbial strains and products thereof have been used for the degradation of environmental contaminants (Kumar et al., 2021). Easy availability, high energy efficiency, lack of toxic residues, and cost effectiveness have made microbes a preferable resource for environmental management. Biosurfactants are one of the emerging microbial biomolecules widely used as lubricants, foaming softeners, dye fixers, emulsion makers, and dispersion stabilizers in various fields, including agriculture, pharmaceuticals, and environmental contaminant cleanup industries (Gharaie et al., 2023; Inès et al., 2023). In general, the term “biosurfactant” refers to biocompatible surface-active molecules with amphiphilic properties and hydrophilic and hydrophobic segments. Usually, the hydrophobic ends are composed of long fatty acids, while the hydrophilic part may be positively or negatively charged or of an amphoteric nature (Farias et al., 2021).

**FIGURE 1:**
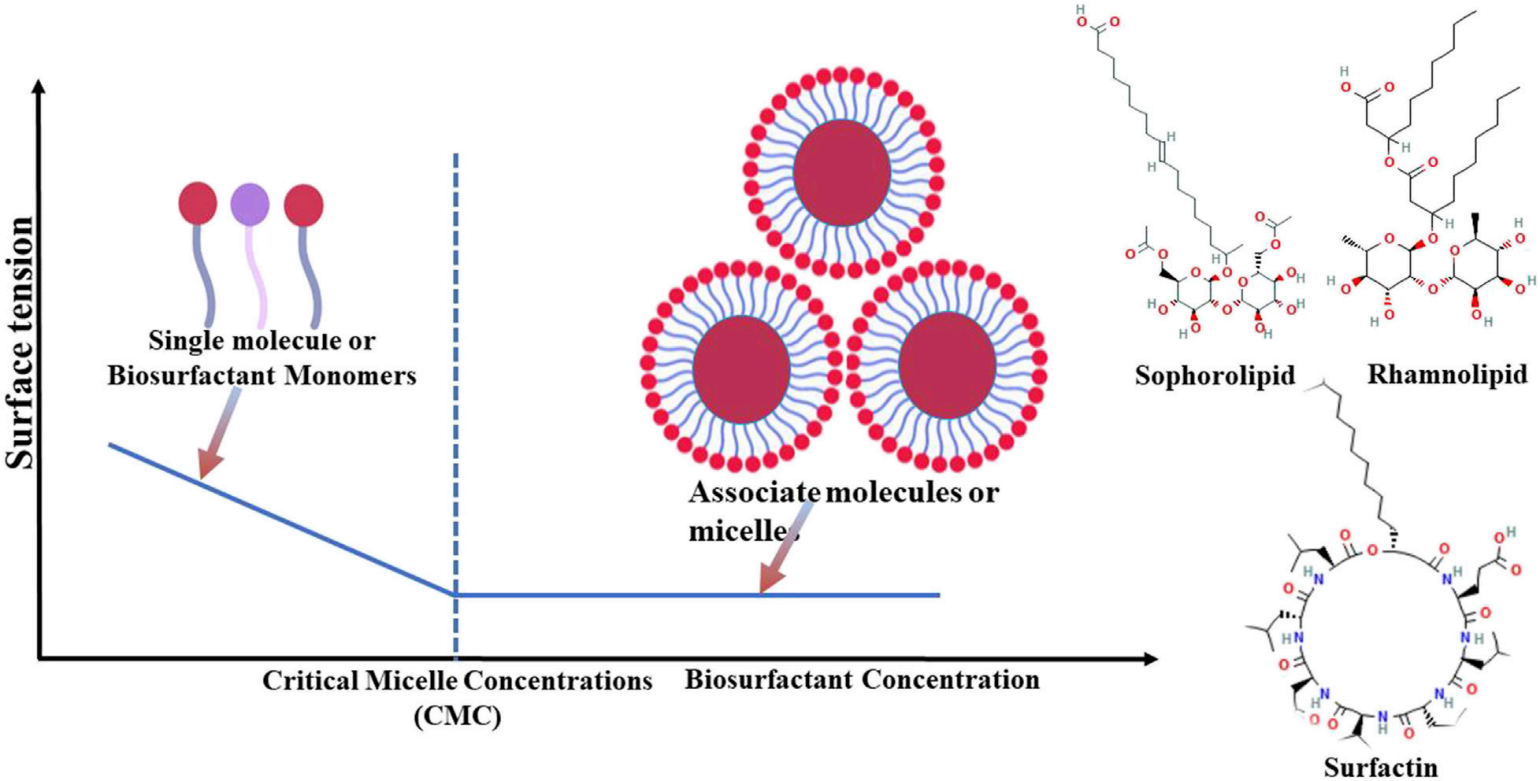
Representation of biosurfactants with their actions for critical micelle concentration and formation of biosurfactant monomers.

Biosurfactants can enhance interaction between different surfaces by forming micelles (Johnson et al., 2021), recognized for effectively reducing surface tension at air–water or oil–water interfaces (Ribeiro et al., 2020; Luz et al., 2023). When surfactants are introduced into systems comprising oil and water or water and air, surface tension is lowered. Beyond a certain concentration threshold, referred to as the critical micelle concentration (CMC) (Abooali and Soleimani, 2023), surfactants start to self-assemble into micelles (Figure 1). Micelle formation is a key mechanism facilitating a reduction in surface tension by biosurfactants (Patel et al., 2023). These properties enable them to effectively reduce interfacial tension between immiscible phases (Kumar et al., 2021).

Recently, biosurfactants synthesized by microorganisms like bacteria and yeasts have garnered considerable attention for environmental contamination management (Kashif et al., 2022). For instance, Al-Marri et al. (2023) reported a strain of Marinobacter sp., isolated from an oil and gas industrial waste site, that had the ability to produce biosurfactants with a promising role in the biodegradation of hydrocarbons. Similarly, Bellebcir et al. (2023) reported the strain of *Streptomyces thinghirensis* as having considerable potential for hydrocarbon bioremediation. A list of biosurfactants produced by different microorganisms is presented in Table 1.

**TABLE 1:** Biosurfactant-producing microbes and their various applications in environmental management.

Microbes	Biosurfactant types	Application	References
*Pseudomonas aeruginosa* (ATCC 10145)	Rhamnolipids	Bioremediation of hydrocarbons, heavy metals, and organic pollutants in soil and water	Wadekar et al. (2012)
*Pseudomonas aeruginosa*		Emulsified and removed vegetable oils and hydrocarbons	Pérez-Armendáriz et al. (2019)
*Pseudomonas aeruginosa* 47T2 NCIB 40044		Dye solubilization and removal from soil and wastewater	Haba et al. (2000)
*Pseudomonas aeruginosa* D		Removal of metals from soil	George and Jayachandran (2013)
*Pseudomonas aeruginosa* MTCC7815		Biocontrol against plant pathogens and efficient removal of pesticides	Sharma et al. (2019)
*Pseudomonas desmolyticum* NCIM-2112		Gasoline and dye degradation	Jadhav et al. (2011)
*Rhodotorula mucilaginosa* SP6		Hydrocarbon emulsification for contaminated areas	Loeto et al. (2021)
*Pseudomonas aeruginosa*		Microbial-enhanced oil recovery	Câmara et al. (2019)
*Lysinibacillus sphaericus* strain IITR51		Aliphatic and aromatic hydrocarbons	Gaur et al. (2023)
*Lactococcus lactis*	Glycolipids	Inhibiting fungal and bacterial populations in soil and detoxification of heavy metals and mycotoxins	Saravanakumari and Mani (2010)
*Lactobacillus* sp.		Bioremediation of oil-contaminated sites	Elshikh et al. (2016)
*Shewanella algae* B12		Remove diesel oil	Gharaei et al. (2022)
*Marinobacter* sp.		Hydrocarbon degradation	Al-Marri et al. (2023)
*Acinetobacter junii* B6	Lipopeptides	Removal of hydrocarbons and detergents	Ohadi et al. (2020)
*Acinetobacter* sp.		Enhanced oil recovery, metal mobilization, and bioremediation of organic contaminants	Deshmukh and Kathwate (2022)
*Kurthia gibsonii* KH2		Bio-decolorization and biodegradation of industrial textile wastewater	Nor et al. (2021)
*Bacillus mojavenis* A21		Efficiently remove dye	Ayed et al. (2019)
*Bacillus licheniformis* V9T14		Eradication of *E. coli* CFT073 biofilm	Rivardo et al. (2011)
*Micromonospora marina*		Bioremediation and biocontrol against pathogenic fungus	Ramalingam et al. (2019)
*Brevibacillus brevis* KN8(2)		Effective bio-fungicide in plant disease management	Krishnan et al. (2019)
*Bacillus amyloliquefaciens* SR1		Antimicrobial activity against fungal plant pathogens, viz., *Rhizoctonia solani*, *Alternaria solani*, *Sclerotium rolfsii,* and *Fusarium oxysporum* under *in vitro* conditions	Nanjundan et al. (2019)
*Bacillus pseudomycoides* BS6		Bioremediation in petroleum-contaminated sites	Li et al. (2016)
*Streptomyces* sp. DPUA1566		Pesticide removal and biocontrol for plant pathogens	Santos et al. (2019)
*Corynebacterium xerosis* NS5		Emulsifying and anti-biofilm activity	Dalili et al. (2015)
*Bacillus licheniformis* L20		Crude oil degradation	Liu et al. (2021)
*Serratia marcescens* UCP 1549		Decrease surface tension, emulsify oil, and improve water solubility	Araújo et al. (2019)
*Starmerella bombicola*	Sophorolipids	Enhanced oil recovery and oil spill remediation	Shah et al. (2017)
*Torulopsis petrophilum*, *Torulopsis bombicola*, and *Torulopsis apicola*		Removal of heavy metals from sediments	Whang et al. (2008)
*Candida bombicola* ATCC 22214		Biodegradation of oils and bioremediation of heavy metals in contaminated soils	Celligoi et al. (2020)
*Starmerella bombicola* (ATCC 22214)		Removal of metals and pesticides from soil	Wadekar et al. (2011)
*Candida tropicalis*		Degradation of diesel oil	Chandran and Das (2012)
*Candida bombicola*		Bioremediation of hydrocarbons	Daverey and Pakshirajan (2009)
*Rhodotorula mucilaginosa*		Crude oil biodegradation	Kazemzadeh et al. (2022)
*Starmerella bombicola*		Household cleaning	Van Bogaert et al. (2013)
*Bacillus velezensis* KLP2016	Surfactins	Emulsification index against benzene, pentane, cyclohexane, xylene, n-hexane, toluene, and engine oil	Meena et al. (2021)
*Bacillus nealsonii* S2MT		Remediation of soil contaminated with heavy engine oil	Phulpoto et al. (2020)
*Bacillus subtilis*		Enhanced oil recovery, bioremediation of oil spills, and wastewater treatment	Sen (2008)
*Aeromonas hydrophila* RP1	Glycolipopeptides	Hydrocarbon degradation from soil and water	Pandey et al. (2022)

**FIGURE 2:**
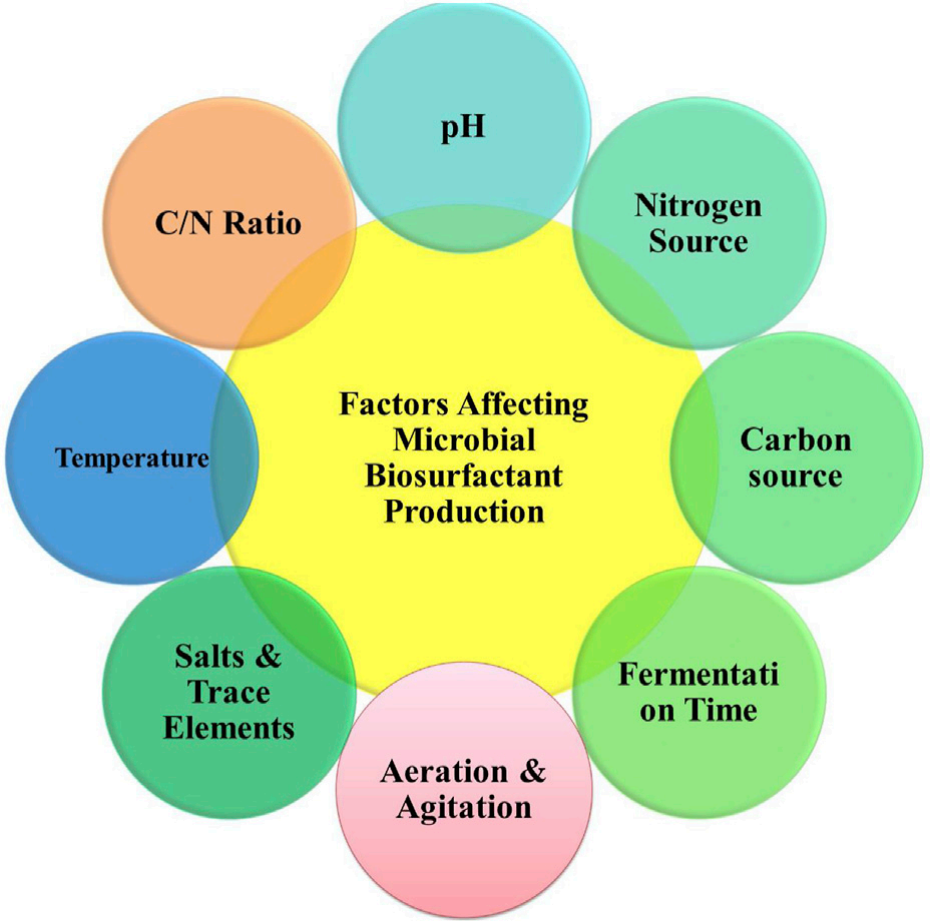
Biosurfactants and their functional roles.

Biosurfactants have been used in different fields, including agriculture, neutraceuticals, pharmacology, cosmetics, and environmental contamination management (Figure 2) due to their ecofriendly and multifunctional properties, thus positioning them as the surfactants of the future (Karnwal, 2023). Microbially produced surfactants offer key advantages like enhanced temperature tolerance, stability at varying pH levels, lower toxicity, and better degradation potential than chemically synthesized surfactants (Karnwal, 2023).

The present review has briefly discussed the current advancements in microbially synthesized biosurfactants, factors affecting production, and the application in remediation of environmental contaminants of hydrophobic nature. In addition, the latest aspects of the circular bio-economy, generating biosurfactants from wastes, and the global economic aspect of biosurfactant production are discussed.

## 2 Role of microorganisms in biosurfactant production

In the recent past, various microbes, including bacteria, fungi, and yeast strains, have been used to synthesize biosurfactants. To achieve optimal biosurfactant production, the optimization of different parameters is required. Various factors like the choice of microbial strains, the additives used in the growth medium, the substrate, and other intrinsic and external factors like temperature, growth conditions, and pH have been considered for the efficient production of biosurfactants (Kashif et al., 2022). The selection of the microbial strain is an initial and crucial step during the process of biosurfactant production. Optimum production of biosurfactants has been observed during the exponential or stationary growth stages of microbial cultures, especially with limited nutritional resources. At this stage, microorganisms can synthesize biosurfactants either intracellularly (within the cell) or extracellularly (outside the cell) (Kashif et al., 2022). Previously, authors have reported biosynthetic pathways and genetic regulation of biosurfactant-producing microorganisms (Sarubbo et al., 2022). Note that biosurfactants derived from different microorganisms can exhibit variations in their composition and functionality. The properties and classification of biosurfactants can vary on the basis of the origin or methods employed during microorganism isolation. Microorganisms isolated from contaminated environments exhibit a unique attribute to degrade specific chemical pollutants as they can use contaminants as an energy source, thereby allowing them to thrive in harsh environmental conditions (Yesankar et al., 2023). Optimization of growth conditions, isolation methods, and selection of potential microbial strains is crucial for harnessing the potential of biosurfactants for diverse applications ranging from environmental remediation to industrial processes.

The current research focuses on identifying and characterizing microbial strains with superior biosurfactant-producing capabilities (Schultz and Rosado, 2020). Additionally, the choice of substrate, such as the carbon source provided to the microorganisms, can significantly impact biosurfactant production. Some microorganisms have been reported to exhibit enhanced biosurfactant production after exposure to specific substrates, while others may prefer alternative carbon sources (Karnwal, 2023). Therefore, tailoring the growth medium to suit the specific needs of the chosen microbial strain is essential for maximizing production. Moreover, genetic modification and synthetic biology have opened up new opportunities to engineer microorganisms in view of enhancing the quality and quantity of biosurfactants (Yesankar et al., 2023).

Although the exact physiological mechanisms behind biosurfactants are not fully understood, it is believed that biosurfactants enhance cellular motility, promote biofilm formation, and improve nutrient uptake from hydrophobic substrates (Yesankar et al., 2023). However, to gain a comprehensive understanding of biosurfactant molecules, it is crucial to develop efficient and accurate methods for isolation and screening of microbial strains (Karnwal, 2023). Numerous studies have reported biosurfactants produced by microbial strains. For instance, rhamnolipid synthesized by the bacterium *Pseudomonas aeruginosa* has been extensively studied and is reported to reduce the surface tension and enhance the emulsification of hydrophobic compounds (Conceição et al., 2020). Other classes of biosurfactants, such as glycolipids and lipopeptides, have also been reported for their effectiveness in reducing surface tension and enhancing the emulsification of hydrophobic pollutants (Kashif et al., 2022). A list of potential microbes synthesizing biosurfactants is presented in Table 1.

The functional behavior of biosurfactants to enhance the availability of hydrophobic compounds and improve microbial activities in challenging environments has garnered significant attention in both scientific and industrial communities (Karnwal, 2023). These versatile compounds have found diverse applications in areas such as bioremediation, agriculture, and healthcare, highlighting their importance. Continued research on biosurfactants, including the exploration of new microbial strains and improved production methods, therefore, promises to unlock their full potential for addressing environmental and industrial challenges.

## 3 Classification of biosurfactants

**FIGURE 3:**
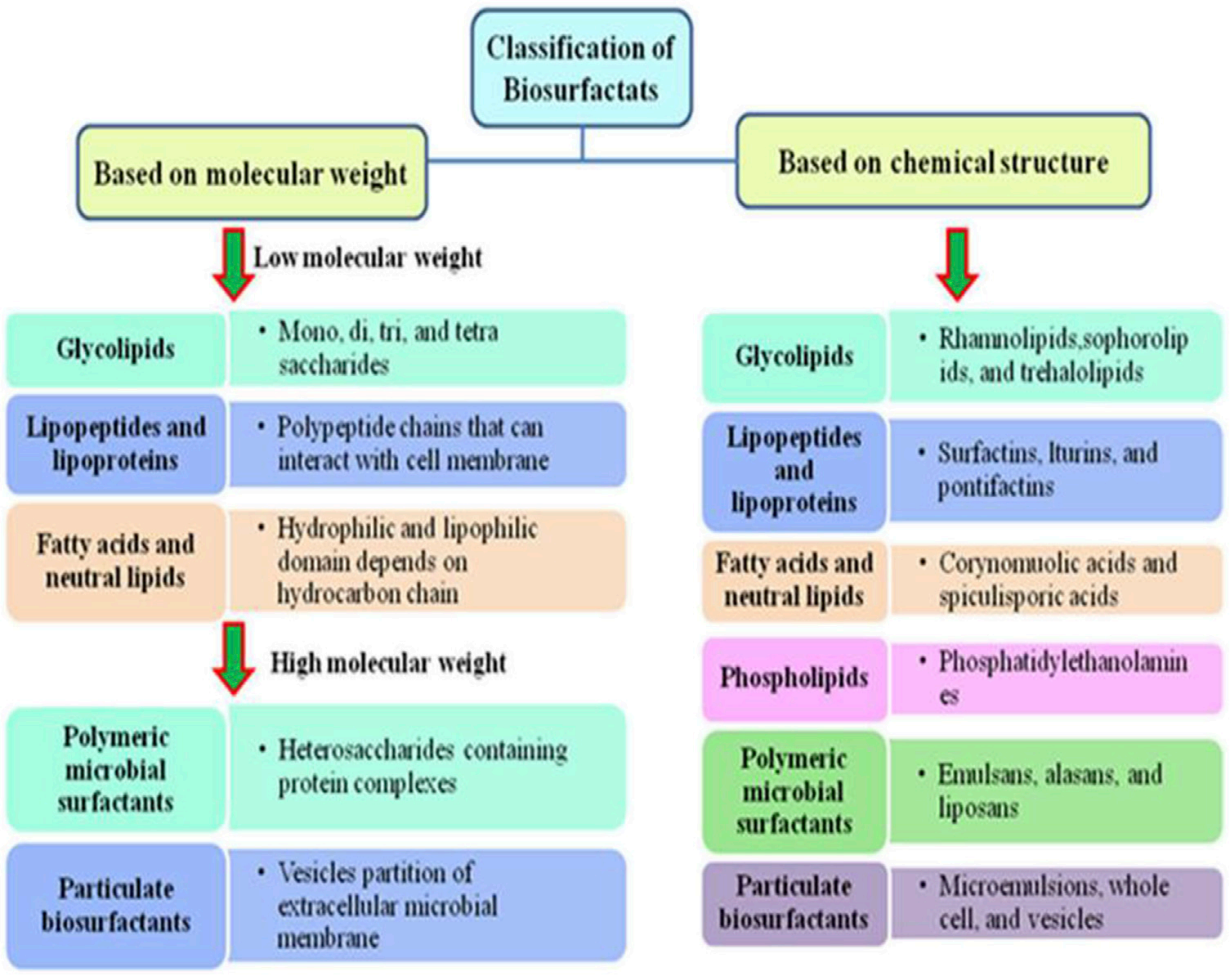
Classification of biosurfactants.

Biosurfactants are commonly classified according to their molecular weight, CMC, microorganisms responsible for their production, and their mechanism of action. In general, biosurfactants can be classified into two groups based on molecular weight. The first group is high molecular weight (HMW) biosurfactants, which are typically composed of intricate biopolymers like polysaccharides and lipopolysaccharides, with a diverse assortment of biopolymers (Hassanshahian et al., 2020). For instance, some HMW biosurfactants, like rhamnolipids synthesized by the strain *Pseudomonas aeruginosa*, have shown promise in applications related to enhanced oil recovery due to their ability to form stable emulsions with hydrocarbons (Chafale and Kapley, 2022). The second group is low molecular weight (LMW) biosurfactants like glycolipids and lipopeptides, which are known for their simpler chemical structures and applied in diverse fields. For example, glycolipids like sophorolipids produced by yeasts have been explored for their emulsification and antimicrobial properties, thereby making them a potential candidate for use in various formulations (Liepins et al., 2021). It has been assumed that large molecular-weight biosurfactants excel in producing stable emulsions, while low molecular-weight biosurfactants produced by microorganisms are efficient at lowering surface tension (Jahan et al., 2020). Details of these classifications are presented in Figure 3.

## 4 Factors influencing the generation of biosurfactants

**FIGURE 4:**
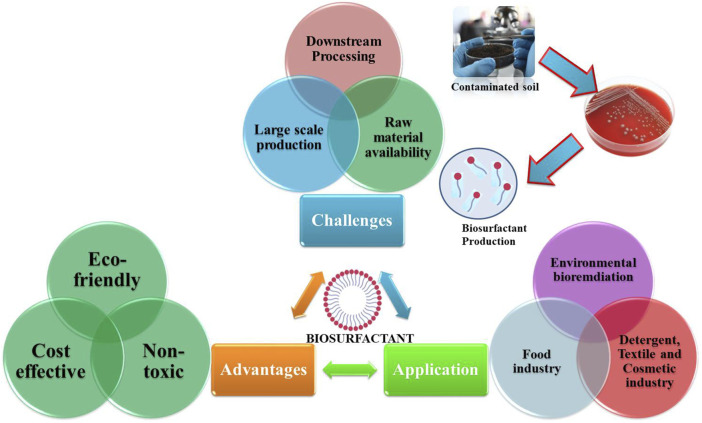
Factors affecting biosurfactant production.

Developing, identifying, and characterizing microbial strains are the initial steps in biosurfactant production. However, to ensure successful biosurfactant production, consistent growth conditions and energy sources such as carbon and nitrogen are required (Sarubbo et al., 2022). The factors influencing the generation of biosurfactants are presented in Figure 4.

Scaling to industrial-level production of biosurfactants requires careful consideration of methodology, chief energy resources, substrate selection, and recovery processes (Jimoh and Lin, 2019). As microbes require different energy sources for their growth, their optimal concentration must be known for increased biosurfactant production (Karnwal, 2023). Previous studies have reported different energy resources for biosurfactant production (Kumar et al., 2021; Kanaoujiya et al., 2022). Production expenses are heavily dependent on raw material supply; however, conventional and ready-made sources of carbon and nitrogen are too expensive for industrial production (Priyani and Munir, 2021).

Significant progress has been made in biosurfactant production, and researchers have continuously examined and tested different energy sources. Studies have used different chief C and N sources either singly or in conjugation with well-established energy sources like glucose and NH_4_^+^ NO_3_^−^. (Agarwal and Sharma, 2009; Bajelani et al., 2023). Such resources include residual waste products, such as food industry waste products, oil wastes, and agro waste. Utilization of such energy resources is cost-effective and reduces production costs by a significant percentage (Santos et al., 2023b; dos Remedios Araújo Vieira Neta et al., 2023; Mallik and Banerjee, 2022; Sarubbo et al., 2022).

In this context, various authors have reported different low-cost alternative sources of carbon and nitrogen to efficiently synthesize biosurfactants. For example, Santos B. L. P. et al. (2023) used pineapple peel as a substrate to produce biosurfactants from the *Bacillus subtilis* strain. dos Remedios Araújo Vieira Neta et al. (2023) evaluated brewer’s spent grain as a carbon source to produce glycolipid biosurfactant from the yeast strain *Rhodotorula mucilaginosa* LBP5. The strain produced a significant amount of biosurfactants having a critical micelle concentration equivalent to 1.5 g/L. Soyuer and Bilen Ozyurek (2023) used low-cost potato waste water as a substrate to produce the biosurfactants from the *Bacillus subtilis* strain. Safari et al. (2023) used rice bran oil as a substrate to produce rhamnolipids from *P. aeruginosa*. Shahabi Rokni et al. (2022) evaluated and optimized sugar beet molasses and other carbon sources for the efficient production of biosurfactants. In a similar fashion, Bouassida et al. (2023) evaluated the different concentration of glucose and their impact on biosurfactant production. Hentati et al. (2019) reported 50 mg L^−1^ of biosurfactants produced by strain *Bacillus stratosphericus* using leftover frying oil as an inexpensive supply of carbon.

## 5 Physical factors involved in biosurfactant production

The synthesis of biosurfactants also depends upon several physical factors like temperature, pH, rate of rotation, and culture growth duration; these factors directly influence microbial growth (Ilori et al., 2005; Al-Bahry et al., 2013). Therefore, physical variables such as the temperature, media, pH, and rate of rotations have been optimized. The temperature largely influences microbial growth, and each of the microbial species showed optimum growth at a particular temperature. For example, the production of glycolipids biosurfactant from *candida* sp. was reported at the temperature of 27°C–30°C (Santos et al., 2016). However, several studies reported an optimum temperature range of 22°C–30°C for biosurfactant production from the bacterial strains isolated from the open ocean (Twigg et al., 2018; Tripathi et al., 2019).

An optimum pH range is also required for biosurfactant production from the microbial species. According to Jimoh and Lin (2019), the optimum biosurfactant recovery for the pH ranges generally observed in the previous studies was between 5.7 and 7.8. Auhim and Mohamed (2013) investigated *Azotobacter chroococcum’s* ability to produce BioS under controlled nutritional and environmental conditions and found that maintaining a pH of around 7 resulted in the highest surface tension reduction of 68% and emulsification index (EC24). Souza et al. (2018) reported 10 g L^−1^ of biosurfactant produced by the strain *Wickerhamomyces anomalus* using olive oil as an energy source under optimal growth conditions. The surfactant showed stability even at 121°C, a salinity concentration of 300 gL^−1^, and pH ranging from 6 to 12. Chooklin et al. (2023) reported the bioremediation potential of glycolipid biosurfactant-producing strain *Bacillus oceanisediminis*. The authors reported an emulsification power of glycolipid equivalent to 65% and a surface tension of 22.67 mN/m after 60 h of cultivation. The proper aeration of the culture is also required for optimum production. The proper aeration at the rotatory shaker directly influences the growth of aerobic microorganisms. The optimum production of biosurfactants was reported at the rotational speed between 50 and 250 rpm (Oliveira et al., 2009; Silvia et al., 2023). However, it has also been reported that faster rotation favors more biosurfactant production (Santos et al., 2016; Twigg et al., 2018).

The incubation time of the biosurfactant-producing microorganisms also plays a crucial role in the synthesis and recovery of biosurfactants. The incubation time varies among the different microbial strains due to different growth rates and growth stages (Santos et al., 2016). A duration of 18–48 h is considered optimum, but in several cases, the incubation time reported is 88–120 h (Felse et al., 2007; Campos et al., 2019). Similarly, the size of the inoculum also plays a significant role in biosurfactant production. In the previous study, the author reported that higher cell density of microbes favors optimum biosurfactant production (Twigg et al., 2018).

## 6 Role of microbial surfactants in environmental management

Diverse microbial entities are known to play an important role in the successful elimination of both organic and inorganic contaminants affecting environmental homeostasis. Microbial processes and products are of considerable interest in the sequestration of individual and mixtures of contaminants. Microbially synthesized surfactants are described as multifunctional candidate molecules with promising potential in environmental management (Fenibo et al., 2019). To date, varied microbial surfactants of prokaryotic as well as eukaryotic origin and low to high molecular weight have been registered (Lang, 2002). In general, the biologically produced surfactants are of a glycolipid nature, possessing sugars like rhamnose and trehalose. The role of microbially produced surfactants in the remediation of environmental contaminants is presented in the following sections.

### 6.1 Heavy metal and metalloid remediation

Contamination with hazardous heavy metals and metalloids originating from different natural and anthropogenic sources is an increasing concern to human health and the natural environment worldwide (Singh et al., 2021a; Singh et al., 2021b). Important heavy metals and metalloids that are an increasing risk to terrestrial and aquatic environments include nickel, zinc, copper, cadmium, aluminum, chromium, arsenic, and mercury. As the presence of metal contaminants above the threshold limit has a detrimental impact on exposed life forms (Gupta et al., 2021; Paithankar et al., 2021; Zamora-Ledezma et al., 2021), their elimination from affected sites using sustainable approaches is imperative. In this connection, the contribution of microbially synthesized surfactants in the ecofriendly remediation of heavy metal and metalloid contaminated sites is quite promising (Wang and Mulligan, 2009; Rastogi et al., 2021). The heavy metal sequestration potential of surfactant-producing *Pseudomonas* sp. CQ2 was recently demonstrated by Sun et al. (2021). The oilfield-isolated *Pseudomonas* with maximum biosurfactant production determined as 40.7 g/L exhibited 8.7%, 65.7%, and 56.9% greater removal of cadmium, copper, and lead, respectively, than chemically synthesized surfactants, suggesting application in bioremediation of contaminated sites. The study showed the involvement of the carboxyl group of biosurfactants in heavy metal chelation and subsequent elimination. The rhamnolipid surfactant produced by *Pseudomonas aeruginosa* strain in the sequestration of lead and mercury from sediment samples has recently been reported (Chen et al., 2021). The biosurfactant at 43.73 mg L^−1^ concentration was 62.5% and 50.2% more effective in removing lead and mercury, respectively, than the common surfactant sodium dodecyl sulfate. The alteration in sediment characteristics after the application of surfactant was noted as an important mechanism responsible for metal removal. Approximately three-fold enhancement in cadmium removal after supplementation of 4 mmol kg^−1^ biosurfactants, namely, rhamnolipids and saponins, was reported by Mekwichai et al. (2020). The rhamnolipid favored greater uptake of cadmium than saponin. Investigation into the capability of rhamnolipid biosurfactant in combination with a glutamic acid derivative for the elimination of heavy metals from sludge waste using an electrokinetic approach was presented by Tang et al. (2017). The synergistic action of biosurfactant and glutamic acid derivative led to enhancement in the removal of copper, zinc, chromium, lead, nickel, and manganese from 60% to approximately 90% during the treatment process. The glycolipid biosurfactant secreting bacterial strain *Burkholderia* sp. Z-90 recovered from sewage sludge has demonstrated effectiveness in the removal of zinc, lead, manganese, copper, cadmium, and arsenic (Yang et al., 2016), ranging from 24.1% to 44%, with minimum removal observed for copper. The interaction of soil minerals with bacteria and the complexation of metal with biosurfactant released was ascribed as the plausible mechanism for the elimination of targeted contaminants. The study indicated the influence of metal speciation on biosurfactant-assisted removal. The amount of biosurfactant synthesized was quite low, thereby restricting broad-scale application for remediation of metal and metalloid contaminated sites.

### 6.2 Pesticide remediation

Increased demand for agricultural products because of the increasing human population, along with the huge postharvest losses of crop produce, has increased the application of diverse hazardous pesticides. The continuous agricultural application of different pesticides has badly affected the aquatic and terrestrial environments (Barbieri et al., 2021; Bijlsma et al., 2021; Egbe et al., 2021; Pérez et al., 2021). The employment of microbially produced surfactants can be viewed as a sustainable tool for the substantial removal of pesticides without compromising the characteristics of the natural environment. Isolation of biosurfactant synthesizing bacteria identified as *Pseudomonas* and *Rhodococcus* causing the degradation of chlorpyrifos has been described recently (Lamilla et al., 2021). The glycolipid and glycopeptide nature of produced biosurfactants were suggested by chemical analyses, and one of the surfactants produced by *Pseudomonasrhodesiae* strain 4 was illustrated to improve the degradation of pesticide by more than 10%. Pesticides with an increased hydrophobic nature could be difficult to treat because of lesser solubility and reduced bioavailability. Increasing the solubility of hydrophobic pesticides using the anionic rhamnolipid biosurfactant, which is stable at a broad range of pH, temperature, and salt and is produced by metal-resistant *Lysinibacillus sphaericus* strain IITR51, is described by Gaur et al. (2019). The introduction of 90 mg/L microbially produced surfactant was observed to induce the dissolution of endosulfan and hexachlorocyclohexane, ranging from 1.8 to 7.2 fold. Biosurfactants are documented to increase the solubility of hydrophobic pesticides (García-Reyes et al., 2018). The experimental investigation suggested an enhancement in the solubility of pesticides, including endosulfan and methyl parathion, after supplementation of biosurfactant of glycolipid nature originating from *Pseudomonas* sp. strain B0406. Another study on the application of *Pseudomonas* sp. (ChlD)-derived rhamnolipid biosurfactant equivalent to 0·1 g L^−1^ has demonstrated the degradation of pesticide chlorpyrifos by nearly 100% (Singh et al., 2009) after 120-h incubation as confirmed by gas and liquid chromatography. In this connection, the isolation of newer bacterial strains with the simultaneous ability of pesticide degradation and biosurfactant production could be a viable approach for field application to manage organic contaminant-affected sites. The basic mechanism pertaining to improved biological degradation of hydrocarbons lies in 1) accelerated solubility of the contaminant utilized as the substrate 2) and surfactant-assisted increment in microbial cell surface hydrophobicity, facilitating the interaction with the contaminant (Mulligan and Gibbs, 2004). Moreover, the presence of a biosurfactant resulted in an increased surface area of less-soluble contaminants due to the diminution of surface as well as interfacial tension, leading to the increased mobilization and biological availability of targeted contaminants.

### 6.3 Petroleum, hydrocarbon, and oil contaminant remediation

Oil spills can destroy environmental quality, making terrestrial and aquatic environments unfavorable for different life forms (Samanta et al., 2002; Philibert et al., 2019; Wei et al., 2020). The present-day physico-chemical technology for the remediation of petrochemicals, hydrocarbons, and oil spills is expensive and detrimental to natural environmental characteristics. Therefore, ecofriendly routes, especially microbial ones, should be developed for the successful elimination of contaminants. Surfactant-producing microorganisms are known for the degradation of petrochemicals, hydrocarbons, and oil contaminants. The action mechanism of the biosurfactant molecules in the degradation of petrochemicals or hydrocarbons is a complex process. The use of biosurfactants generally reduces the surface and interfacial tension between the water and hydrocarbon molecules, resulting in increased emulsification and dispersion of hydrocarbons in water. In addition, the biosurfactant molecules form micelles over the hydrophobic petrochemicals or the hydrocarbon molecules, which further increases the solubility of these hydrophobic contaminants in water. These processes increase the bioavailability and solubility of hydrophobic pollutants and are also crucial for microbial degradation (Desai and Banat, 1997).

Some studies have reported that the biosurfactant molecules facilitate the formation of biofilms over the hydrocarbon surfaces. Biofilms are microbial communities embedded in a self-produced extracellular matrix that enhances the stability and efficiency of microbial consortia in degrading pollutants (Zhang and Miller, 1994). It has also been reported that some biosurfactant molecules interact directly with hydrocarbons, initiating chemical changes that make them more susceptible to microbial attack. This can involve processes like oxidation or the breaking of complex hydrocarbon chains into simpler compounds (Rahman et al., 2002).

Previously published reports have reported the role of microbial-synthesized biosurfactants in the bioremediation of petrochemicals, hydrocarbons, or oil spills. Pandey et al. (2024) reported the glycolipid surfactant produced by the *Citricoccus zhacaiensis* strains, which showed an effective role in the degradation of petrochemical hydrocarbons. The strain showed ≥95% degradation of hydrocarbon pollutants in a 2-week period. Csutak et al. (2024) reported the role of biosurfactant produced by the yeast strain *Yarrowia lipolytica* CMGB32 in the remediation or degradation of n-hexadecane petrochemical pollutants. Li et al. (2024) also reported the effective role of biosurfactant lipopeptides produced by *Raoultella planticola* in the degradation of n-hexadecane-contaminated soil.

The contribution of *Bacillus velezensis* KLP2016 synthesizing a biosurfactant designated as surfactin is noted for the degradation of engine oil (Meena et al., 2021). The presence of biosurfactants at 35°C and 25°C lowered the surface tension of a cell-free medium, pointing toward their application for enhanced biodegradation of engine oil. The media supplementation with different carbon and nitrogen substrates may be tested for further improvement in biosurfactant production and consequent biodegradation. The biosurfactant purified by electrospray ionization coupled with mass spectroscopy revealed a molecular weight equivalent to approximately one kilo Dalton. Recently, the increased remediation of oil sludge based on the composite biosurfactant of lipopeptide (anionic) and sophorolipid (non-ionic) nature employed as a washing agent compared to a single treatment was demonstrated (Bao et al., 2021). Lipopeptide and sophorolipid biosurfactants with a mass ratio of 8:2 exhibited reduced CMC and displayed maximum synergistic action with optimum removal under defined washing conditions of temperature, shaking, treatment time, and sludge to-liquid proportion. In addition, the study also proposed the influence of minerals on oil sludge treatment. The CMC was found to be lower than the standard value. The involvement of *P. aeruginosa* and *Meyerozyma* sp. producing a surfactant with a rhamnolipid (critical micelle concentration 40 mg/L) and sophorolipid (critical micelle concentration 50 mg/L) nature, respectively, in crude oil degradation was presented by Rehman et al. (2021). The synthesized biosurfactants were stable at varying pH, temperature, and salt concentrations and led to the 87% to 91% degradation of petroleum hydrocarbons. The biosurfactants were shown to hold promising avenues in the decontamination of oil-contaminated soils. Lai et al. (2009) registered superior oil remediation activity of biosurfactants, namely, rhamnolipids and surfactin, in contrast to the chemically synthesized surfactants Tween 80 and Triton X-100. At 0.2% content of selected biosurfactants and synthetic surfactants, the percent removal of petroleum hydrocarbon ranged from 35% to 63%, with the maximum removal recorded for biosurfactants and the minimum for synthetic surfactants. The analysis suggested an increase in the removal of total petroleum hydrocarbons with increased surfactant and contaminant concentrations.

In contrast to chemical surfactants, biosurfactants are ecofriendly in nature, cost-effective, biodegradable, less toxic, have a large number of functional groups, and do not produce secondary pollutants (Chen et al., 2011; Mao et al., 2015). Moreover, the biosurfactant could have greater chemical diversity and potential in the cleanup of contaminated sites, implying utilization for extensive bioremediation purposes. However, the microbes used for surfactant production followed by contaminant removal may suffer from challenges imposed by environmental variables like pH, temperature, and nutritional requirements. The changing environmental conditions may considerably influence the survivability of microbes as well as the optimum production of biosurfactants. Moreover, the production of biosurfactants by a given microorganism varies considerably from one species to another, implying the isolation of suitable species for efficient remediation. Therefore, process optimization to facilitate the maximum biosurfactant production and subsequent remediation of the contaminant of interest is necessary for harnessing the potential of diverse microbially originated surfactants.

## 7 Biosurfactant production from waste: a new aspect in the circular bio-economy

In the last two decades, the use of microbial strains for biosurfactant production has been gaining momentum due to its ecofriendly nature, low cost of production, and the easy availability of microorganisms. However, the rate of commercial production of biosurfactants is still very slow due to the high cost of media or the resource materials (Kumar et al., 2021). During biosurfactant production, the optimum growth of microorganisms requires carbon, nitrogen, or energy resources. Although in laboratory conditions, the regular media used for microbial growth does not appear as a hurdle, on a larger or industrial scale, the cost of media or energy resources is a major challenge (Eras-Muñoz et al., 2022; Mgbechidinma et al., 2022).

Currently, research communities are working to find an alternative to the regular media, especially for carbon, nitrogen, or energy resources. Currently, various alternative sources, such as vegetable waste products, agricultural residues, and fruit waste, have been used as alternative media sources for the growth of microorganisms or biosurfactant products (Kumar et al., 2021; Ciurko et al., 2022). Agricultural waste, fruit waste, molasses, sugarcane waste products, sunflower oils, cake bakery products, soybeans, potato peels, etc., have been frequently utilized as an alternative source of the regular media for biosurfactant production. In agricultural waste, rice husk, wheat straw, sugarcane bagasse, and maize cobs are the frequently used alternative sources practiced for biosurfactant production (Kumar et al., 2021; Ciurko et al., 2022; Santos C. V. M. et al., 2023). Optimization of media and the compatibility of microorganisms are priorities for agricultural waste selection (Silva et al., 2024).

The high nutrient contents and rich availability of carbon, nitrogen, protein, and lipid sources in fruit waste offer an alternative or rich media source for biosurfactant production (Kumar et al., 2021; Varjani et al., 2021; Gaur et al., 2022) Generally fruits peels, pulps, and seeds have been considered fruit waste. In recent years, various authors have reported the optimum production of biosurfactants utilizing fruit waste products (Sharma and Pandey, 2020). In current biosurfactant products, various by-products of the sugarcane industries, such as corn, molasses, and sugar beet, have been used due to their high starch and sucrose content, which provide ample supplements for microbial growth (Kumar et al., 2021).

## 8 The latest global economic aspects of biosurfactant production

Despite the broad and environmentally friendly application of biosurfactants, the limited and expensive nature of raw materials hinders biosurfactant production at the industrial level (Saisa-Ard et al., 2013; Henkel et al., 2017). However, the global trend of biosurfactant production increased significantly in 2022, where the market value of biosurfactants was $1.9 billion, and it is expected to reach $3.2 billion by 2032, with a 5.4% compound annual growth rate (CAGR) growth (https://www.alliedmarketresearch.com/biosurfactant-market).

Europe is currently the largest emerging market for biosurfactants, followed by the United States. The demand for biosurfactants is also growing in Asian countries due to increased infrastructure and awareness about the environmental benefits (Singh et al., 2019). Their rise in the European market is due to the demand in the personal care or cosmetics industries. The major uses of biosurfactants have been reported in the detergent industries, followed by personal care and agricultural chemicals in 2022 (https://www.marketsandmarkets.com/). Evonik Industries AG (Germany), Deugan Biosurfactant supplier (China), and Saraya Co. Ltd. (Japan), etc., are the key market players in the industry (https://www.marketsandmarkets.com/).

Approximately 30%–50% % of total biosurfactant production cost is due to the price of raw materials (Saisa-Ard et al., 2013; Kumar et al., 2021). Successfully transitioning waste products, including agricultural waste, sugarcane waste products, and fruit or vegetable waste, as a carbon or energy source in a circular economy is essential for balancing industrial growth, economic development, and environmental protection. This approach also enhances the strategic use of resources (Varjani et al., 2021; Gaur et al., 2022).

## 9 Challenges and opportunities for microbial biosurfactant production

The application of biosurfactants as an alternative to fossil-derived surfactants has the potential to diminish carbon dioxide emissions by 8%, amounting to 1.5 × 10 t (Rocha e Silva et al., 2019; Farias et al., 2021). The reduced carbon emissions underscore the importance of biosurfactants in view of sustainable development goal 13. Biosurfactants are receiving considerable attention from scientists worldwide in the pharmaceutical and oil recovery industries because of their inherent properties to increase the solubility and availability of drugs in an aqueous environment (Das and Rao, 2024; Pannu et al., 2024; Purwasena et al., 2024; Rehman et al., 2024). Recently, a biosurfactant released from the yeast *Scheffersomyces shehatae* has demonstrated the potential for application in cosmetic products (Cedrola et al., 2024). The employment of diverse waste resources of a biological nature for the synthesis of biosurfactants has the potential to restrict the cost of waste disposal (Tang et al., 2023). Thus, the exploitation of waste to support the production of valuable resources strengthens the concept of circular economy (Venkataraman et al., 2024). Furthermore, the successful utilization of waste resources may minimize hazardous impacts on ecosystem integrity.

## 10 Conclusions

Continuously increasing industrial activities release large numbers of contaminants into the environment and severely affect the soil and water ecosystem through the accumulation of chemicals, industrial effluents, oil spills, etc. The toxic nature and long-term persistence of industrial pollutants pose severe health risks to human beings, and their sustainable mitigation is a serious concern of government and policymakers. A wide range of traditional physical and chemical methods have been followed to mitigate the challenges of industrial pollutants. However, pollutants having a hydrophobic nature, such as hydrocarbons, oil spills, and pesticides, are difficult to manage using traditional methods. In this regard, biosurfactants are being considered for degrading contaminants of a hydrophobic nature. Although biosurfactants produced by microorganisms are intrinsically economical, the need for carbon and nitrogen-based energy resources required for microbial growth is costly. Therefore, there is an urgent need to harness alternative energy sources, appropriate microbial candidates, and growth conditions to optimize the synthesis of biosurfactants by microbes in a sustainable and economical way.
